# A Novel Anti-EGFR mAb Ame55 with Lower Toxicity and Better Efficacy than Cetuximab When Combined with Irinotecan

**DOI:** 10.1155/2019/3017360

**Published:** 2019-01-13

**Authors:** Weiyi Qiu, Chang Zhang, Shuang Wang, Xiaoyan Yu, Qiong Wang, Dadi Zeng, Peng Du, Jinling Ma, Yiqiong Zheng, Bo Pang, Yunzhou Yu, Feng Long, Xiaobin Pang, Zhiwei Sun

**Affiliations:** ^1^Antibody Engineering Group, Beijing Biotechnology Institute, 100071 Beijing, China; ^2^Pharmaceutical Institute, Henan University, 475004 Kaifeng, China; ^3^Joinn Laboratories (China), Co. Ltd, 100176 Beijing, China; ^4^301 Hospital, 100853 Beijing, China; ^5^Clinical Laboratory, Guang'anmen Hospital, China Academy of Chinese Medical Sciences, 100053 Beijing, China

## Abstract

To improve efficacy and minimize toxicity of EGFR inhibition treatment, we developed Ame55, a novel anti-EGFR IgG1 with lower affinity to EGFR than cetuximab (C225) from a human phage library. Ame55 had lower bioactivity than cetuximab *in vitro* but similar antitumor efficacy as cetuximab *in vivo*. Moreover, Ame55 was more efficacious than cetuximab in a Lovo cell xenograft tumor model when combined with irinotecan (CPT-11). Ame55 concentrates in the mouse xenograft tumor and has less toxicity than cetuximab in cynomolgus monkeys in an overdose study.

## 1. Introduction

Epidermal growth factor receptor (EGFR) is a transmembrane receptor tyrosine kinase (RTK) and a member of the ErbB (human epidermal receptor (HER)) receptor family. It is an important protein in cell proliferation and tissue development and is widely expressed in normal epithelial tissues, especially skin and digestive tract epithelium. EGFR is also overexpressed in about one third of all human cancers [1, 2], and EGFR-mediated activation of downstream signaling pathways is associated with poor patient outcomes [3]. So, EGFR is an attractive therapeutic target for the treatment of epithelial cancers.

However, the wide expression of EGFR is also the major cause of the side effects of EGFR inhibition drugs. Target-related side effects are still the major obstacle for cancer patients who are undergoing EGFR inhibition treatment. For EGFR-mAbs, patients have suffered with diarrhea, skin disorders, and other symptoms. Indeed, 10% of patients withdraw from treatment because of severe side effects [4]. Thus, reducing affinity of mAbs to EGFR is one consideration to decrease side effects of an antibody drug.

Generally speaking, side effects and potency of anti-EGFR antibodies are usually associated with their affinity, and affinity usually associated with cytotoxicity. As generally accepted, high affinity is commonly accompanied by higher toxicity, while low affinity predicts lower antitumor capacity. To assure adequate tumor inhibition effectiveness, the US Food and Drug Administration (FDA) has approved only three EGFR-targeted mAbs drugs, including cetuximab (C225) [5, 6], panitumumab [7, 8], and necitumumab [9, 10]. These all have high-level receptor-binding affinity and undesirable side effects, such as serious diarrhea and skin rash [4]. Conversely, nimotuzumab (h-R3), which has been approved by the China National Medical Products Administration (NMPA), exhibits relatively low affinity and cytotoxicity [1]. A good efficacy of nimotuzumab on non-small cell lung cancer (NSCLC) indicated that intermediate affinity for EGFR showed a low toxicity profile and allowed its use on long-term chronic treatment [11].

Sometimes, the affinity-efficacy correspondence does not hold true. For example, panitumumab, whose affinity is higher than cetuximab, showed stronger toxicity such as diarrhea and dermal rashes, but there was no clear improvement in potency [12, 13] because of the penetration limit effect [14].

Consequently, exploring new anti-EGFR antibodies with a good balance of affinity-efficacy-toxicity is very urgent for clinical treatment of epithelial cancer. A mathematical model on nimotuzumab predicted that an antibody with intermediate affinity would reach a maximum difference between the area under the curve of tumor and normal tissues [2]. We believe that there must be a critical point between efficacy and toxicity, indicating a good antitumor efficacy but with few side effects. Better drugs are always desired. Specifically, multiple anti-EGFR mAbs are under investigation that target variable EGFR mutation subtypes [15–17] to improve specificity and avoid drug resistance [16, 18, 19]. Some other novel mAbs targeting EGFR are in clinical trials [10, 17, 19–21].

In the present study, we attempted to investigate a novel anti-EGFR antibody with a good balance between affinity and toxicity by screening for reasonable affinity with novel binding sites. To advance innovation in drug development, we developed Ame55, a novel anti-EGFR IgG1 from a human phage library. A series of *in vitro* assays and *in vivo* tests were conducted to explore its affinity, binding specificity, xenograft tumor inhibition, combined efficacy, and general toxicity.

## 2. Materials and Methods

### 2.1. Cell Culture and Reagents

A total of 4 cell lines were used in the current study. The A431 and HaCaT cell lines were purchased from ATCC (Manassas, USA) and Difi, Lovo, and CHO cell lines were purchased from CAS (Chinese Academy of Science, Shanghai, China). All cells were maintained in appropriate medium supplemented with 10% fetal bovine serum (Gibco, Paisley, Scotland) and kept at 37°C with 5% CO_2_ in a humidified air incubator. Fusion protein hFc-EGFR, His-EGFR with the full extracellular domain (L25 to G640), and fully synthetic human scFv phage displayed libraries were constructed by our laboratory [22].

### 2.2. Screening of Fully Synthetic Human scFv and IgG1 Construction and Expression

Phage libraries and scFv screening were performed as previously described by Du et al. [22]. Phage-displayed libraries were prepared according to recombinant phage selection module protocol Cat. #XY-040-00-05 (Pharmacia, Stockholm, Sweden). After 3 rounds of selection, single clones were screened by ELISA with BSA as a negative control. V_H_ and V_L_ genes of immunopositive scFvs were cloned into expression vector pAbG1 using restriction enzyme sites. For heavy chain, these were *Afl*II and *Nhe*I, and for light chain these were *BsrG*I and *Hind*III. Heavy-chain and light-chain expression vectors were used to cotransfect FreeStyle™ 293-F cells (Invitrogen, Carlsbad, CA) for instantaneous expressions. Supernatants containing IgG1 were collected and purified with a Protein A 1 mL column (GE Healthcare, Uppsala, Sweden). Then, a 10% SDS-PAGE reducing gel was used to confirm antibody purity.

### 2.3. Affinity Analysis

#### 2.3.1. Affinity Analysis by Biacore

Affinity assessment between antibodies and recombinant EGFR was measured with calculations from kinetic constants using a Biacore 3000 system. Multicycle kinetics were analyzed. Purified antibodies (1.0 *μ*g/mL) were captured on CM5 chips (GE Healthcare, BR-1000-12, Uppsala, Sweden) with the Human Antibody Capture Kit (GE Healthcare, BR-1008-39, Uppsala, Sweden). Recombinant his-EGFR (ACROBiosystems, EGR-H5222, Newark, DE) at different concentrations (from 3.1 to 100 nM for Ame55 and 0.39 to 100 nM for cetuximab) were passed over the chip. A 3 min association time was followed by a 2–15 min dissociation. After each cycle, the chip was regenerated with glycine HCl (pH 1.5) and borate (pH 8.5). Repeated measurements were performed at 6.25 nM. Then, purified Fc-rEGFR (1.0 *μ*g/mL) was captured on a CM5 chip with the Human Antibody Capture Kit, and Fab fragments with different concentrations (50 to 800 nM for Ame55 and 1.56 to 50 nM for cetuximab) were passed as depicted above. For the binding between the full antibodies and the bivalent hFc-EGFR construction, antibodies with gradient concentrations (1 to 100 nM) were captured on CM5 chips, and purified recombinant Fc-EGFR (0 to 100 *μ*g/mL) were passed over the chip. Experimental data were fitted to a 1 : 1 binding model using Biacore ™ 3000 evaluation software. KD, namely as affinity, constant was calculated from the ratio of the rate constants Koff/Kon.

#### 2.3.2. Affinity Analysis by Cell ELISA

A431 cells were seeded in 96 well-plates, from 0.5 × 10^4^/well to 4 × 10^4^/well, and were cultivated overnight. Then, the antibodies were diluted to 10 *μ*g/mL with PBS containing 2% FBS and serially two-fold diluted for 8 gradients. After 1 h on ice, plates were washed with PBST. HRP-conjugated goat anti-human IgG (Sigma-Aldrich, A0170) was added and incubated for 30 min on ice. After washing, o-phenylenediamine dihydrochloride (OPD) substrate was added, and optical density (OD) was read at 492 nm with 630 nm as a reference using a microplate reader (Thermo Multiskan MK3, Piedmont, SC). The relative binding affinity, defined as the concentration giving the half-maximal A, is an approximation of an antibody's KD.

### 2.4. Competitive Binding Assay

**Figure 1:**
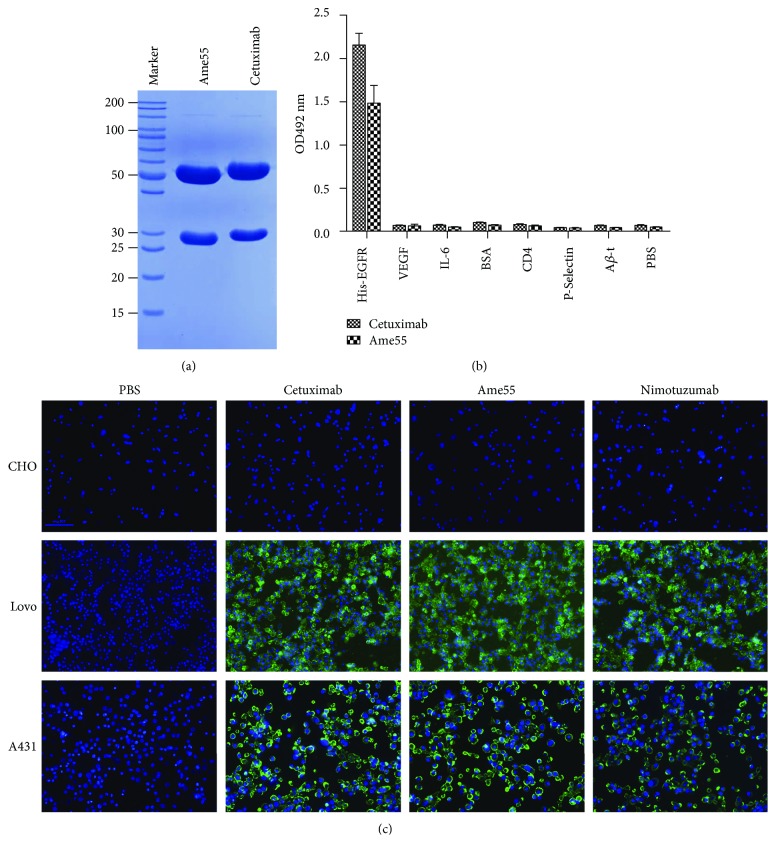
Ame55 binding to EGFR. (a) Analysis of cetuximab and purified Ame55 antibody with 10% SDS-PAGE (reducing) gel. (b) Specific binding of Ame55 and cetuximab to different proteins (10 *μ*g/mL) with ELISA. (c) Binding of cetuximab, Ame55, and nimotuzumab (50 *μ*g/mL each) in CHO (no EGFR), Lovo (moderately expressed EGFR), and A431 (highly expressed EGFR) cell surfaces.

Competitive binding experiments were performed by the Octet QK^e^ System (ForteBio Inc.) to investigate the overlap of the antigenic determinants of Ame55, cetuximab, and nimotuzumab on EGFR Domain III. The SA sensor surface was coated first with 500 nM of the mAbs Ame55 (Figure 1(c), A), nimotuzumab (Figure 1(c), B), or cetuximab (Figure 1(c), C). 200 nM His-EGFR was loaded to saturate binding after washing (by HEPES, pH 7.2). After washing, 500 nM of the second mAbs (Ame55, nimotuzumab, and cetuximab) was added to compete with the binding (Figure 1(c)).

### 2.5. Binding Specificity Analysis

Binding specificity analysis was performed using cell ELISA, solid ELISA, and immunofluorescence assays described as follows.

#### 2.5.1. Solid ELISA

For the binding specificity assay, 200 ng His-EGFR, VEGF, IL-6, BSA, CD4, P-selectin, A*β*-T (all proteins were expressed by our laboratory), and PBS were coated in 96-well plates (Costar 9018, Corning, NY) in PBS buffer and incubated at 4°C overnight. Then, 5% fat-free powdered milk in PBS with 0.1% Tween-20 was used for blocking for 30 min at 37°C. Then, cetuximab and Ame55 were used as primary antibodies and incubated for 1 h at 37°C. After 3 washings, HRP-goat anti-human IgG (ZSGB-BIO, Beijing, China) was used as the second antibody and was incubated for 30 min at 37°C. Plates were developed with OPD, and optical density (OD) was read at 492/630 nm using a 470 microplate reader (Thermo Multiskan MK3, Piedmont, SC).

#### 2.5.2. Immunofluorescence Assay

For the EGFR-antibody binding assay, 10,000 CHO, Lovo, or A431 cells at the exponential phase of growth were seeded on coverslips in 24-well tissue culture plates. After overnight culture, cells were washed with PBS containing 4% paraformaldehyde (Beijing Chemical Works, Beijing, China) and 0.1% Triton X-100 (Sigma, St. Louis, MO) and then were washed with PBS at room temperature. Cells were then blocked with 5% goat serum in PBS for 30 min, washed three times with PBS, and incubated with 50 *μ*g/mL Ame55 or cetuximab, for 2 h at room temperature. PBS was used as the negative control in place of the antibody. Cells were then washed three times with PBS and incubated with FITC-labeled goat anti-mouse IgG (ZSGB-BIO, Beijing, China) diluted at 1 : 200 in PBS for 30 min. Cells were rinsed in PBS three times and visualized under a microscope with 100x zoom (Nikon Xi-80, Tokyo, Japan).

### 2.6. Immunoblotting and Immunostaining

#### 2.6.1. Western Blotting

Cell lysates or tumor tissue lysates were lysed in RIPA buffer containing protease inhibitor cocktail (Roche, Mannheim) and kept on ice for 15 min. Samples were ultrasonicated and centrifuged to obtain supernatant. Immunoblots were performed according to published procedures [19]. Briefly, protein lysates were subjected to sodium dodecyl sulfate-polyacrylamide gel electrophoresis (SDS-PAGE) at 160 V (Bio-Rad) on a 12% gel (CWBIO, China) for 50 min, then transferred to a 0.22 *μ*m nitrocellulose filter membrane (Solarbio, China) at 15 V for 20 min. The membrane was washed in TBS-Tween 20 for 5 min at 25°C, then incubated in blocking buffer for 1 h at 25°C, and then washed in TBS-Tween 20 three more times. The membrane was incubated overnight with primary antibodies at 4°C with gentle shaking and was then washed three times followed by incubation for 1 h at 25°C with the secondary antibody (anti-rabbit IgG, Cell Signaling Technologies, USA). Finally, the membrane was visualized using chemiluminescence (ECL) immunoblotting detection reagents (Thermo Fisher Scientific, USA). Primary antibodies were as follows: antibodies against EGFR (pY1068/1173), AKT, pAKT, ERK, pERK, Grb2, and GAPDH were purchased from Bioworld Technology (Nanjing, China); anti-EGFR antibody (SC-03) was from Santa Cruz (Paso Robles, CA); and HRP-tag anti-M13KO7 antibody was from GE (Uppsala, Sweden).

#### 2.6.2. Immunohistochemical Staining

To assess angiogenesis and cell proliferation in tumors, formalin-fixed paraffin-embedded tumor tissues were immunostained with a monoclonal mouse anti-human PCNA antibody (ZSGB-BIO, China) and S9001 IHC staining kit (ZSGB-BIO, China). After deparaffinization and rehydration, the tissue sections were incubated with 3% hydrogen peroxide in methanol to quench endogenous peroxidase. Sections were blocked for 30 min with goat serum and incubated overnight with primary antibody at 4°C. Then, the sections were washed with PBS and incubated with a biotinylated secondary antibody for 30 min. Last, they were incubated with HRP-conjugated streptavidin for 30 min. Sections were visualized using a diaminobenzidine ZLI-9017 staining kit (ZSGB-BIO, China) and counterstained in hematoxylin.

### 2.7. Nude Mouse Xenograft Tumor Inhibition Assay

Both single (Ame55 or cetuximab) and combined (Ame55 and Cetuximab) treatment were performed in animal tests. For single treatment of the A431 xenograft inhibition assay, female nude mice (*n* = 9/group, 14–17 g) were subcutaneously injected with 5 × 10^6^ A431 cells (100 *μ*L). When tumors reached a mean volume of 150 mm^3^, mice were randomly assigned to receive different concentrations of antibodies and solvent injections: cetuximab (0.25 and 0.5 mg, i.p.), Ame55 (0.25, 0.5, and 1 mg, i.p.), adalimumab (0.25 and 0.5 mg, i.p.), or sterile PBS (i.p.) three times per week for 3 weeks. All of the mice were sacrificed after 24 days. For the combined treatment assay, A431 xenograft mice (*n* = 5/group) were treated with 0.15 mg Ame55 or cetuximab antibodies twice per week, and 30 ng irinotecan was given once per week. Mice were sacrificed after 12 days. Lovo xenograft mice (*n* = 5/group) were treated with 0.5 mg Ame55 or cetuximab antibodies twice per week and 30 ng irinotecan once per week and were sacrificed after 53 days of treatment. Tumor volumes were measured before each treatment [volume = *π*/6 × length × (width)^2^]. Mice were anesthetized and sacrificed. Weights and tumor volumes were recorded and compared. Tissues were lysed, and Western blotting was done as described above. Data were expressed as percent inhibition of tumor growth.

### 2.8. Safety Test in Cynomolgus Monkeys

Ame55 was administrated to 2 cynomolgus monkeys (1 male and 1 female) via intravenous infusion at a total dose of 300 mg/kg twice with a 2.5-hour interval (150 mg/kg/interval). The animals were observed for 4 hours after dosing and then twice a day (AM and PM) for the next 14 days. During the observation, the body weights were recorded before treatment and on the 8th day and 15th day after the treatment. The body temperature, electrocardiogram, hematology, coagulation, and clinical chemistry parameters were measured on the pretreatment day and on the 2nd, 4th, 8th, and 15th days post-treatment. The animals were euthanized and necropsied for gross examination on the 15th day post-treatment.

### 2.9. Statistical Analysis

Data are presented as means ± standard deviation (SD) and were analyzed using a Student *t* test or 2-way ANOVA (*p* < 0.001 was considered statistically significant).

## 3. Results

### 3.1. Ame55 Development and Validation

A fully synthetic human scFv library containing up to 1.35 × 10^10^ clones [23] was used for screening with fusion protein hFc-EGFR as an antigen. Three selection rounds were performed, and positive clones were identified via semiquantitative ELISA. Among these, 144 positive clones were sequenced. Of these, 95% shared the same sequence with the #55 clone which was sequenced first. The variable region of light- or heavy-chain genes of the scFv #55 were, respectively, cloned into expression vectors pABL and pABG as previously described by Du et al. [22]. The IgG1 of #55 (named Ame55) was expressed in HEK293T cells and purified. Ame55 was identified via SDS-PAGE (Figure 1(a)), which depicted a protein with ~50 kDa heavy chain and a 28 kDa light chain, all slightly smaller than those of cetuximab [6]. All these data indicated that a new monoclonal anti-EGFR had been selected.

### 3.2. Specificity and Binding Activity of Ame55

The specific binding of Ame55 to recombinant his-EGFR or to the natural form (A431 and Lovo surface EGFR) was confirmed with ELISA and immunofluorescence. The results of the ELISA (Figure 1(b)) demonstrated that Ame55 exhibited binding of His-EGFR which was similar to the binding of Erbitux (cetuximab) to His-EGFR. The binding of Ame55 with EGFR was much stronger than the binding of Ame55 with VEGF, IL-6, BSA, CD4, P-selectin, A*β*-T, and the blank control (PBS). Immunofluorescence results (Figure 1(c)) showed that Ame55 could bind to the Lovo cell surfaces (with moderately expressed EGFR) and especially to the A431 (highly expressed EGFR) cell surfaces, but not to the CHO (no EGFR) cell surfaces. Compared to C225 and HR3, a larger number of Ame55 molecules bound to A431. Meanwhile, the binding force of Ame55 was measured.

To identify binding affinity of mAbs to EGFR, two fusion proteins (His-EGFR and hFc-EGFR) and two forms of mAbs (IgG or Fab of cetuximab and Ame55) were used in Biacore tests. Binding avidity was performed via cell ELISA tests using A431 cell surface EGFR binding to cetuximab, Ame55, or nimotuzumab. The results illustrated that, in binding to EGFR, Ame55 has significantly lower monovalent affinity than cetuximab (468 nM vs. 1.24 nM or 931 nM vs. 1.39 nM), lower bivalent affinity (avidity) than cetuximab [0.23 nM vs. <0.1 nM (below the detectable limit)] (Table 1, Supplemental Table 1), and lower binding activity than cetuximab (1.7 nM vs. 0.46 nM). In addition, Ame55 has higher binding activity to EGFR than nimotuzumab (Table 1).

### 3.3. The Epitopes of Ame55 Are Different from That of Cetuximab and Nimotuzumab

**Figure 2:**
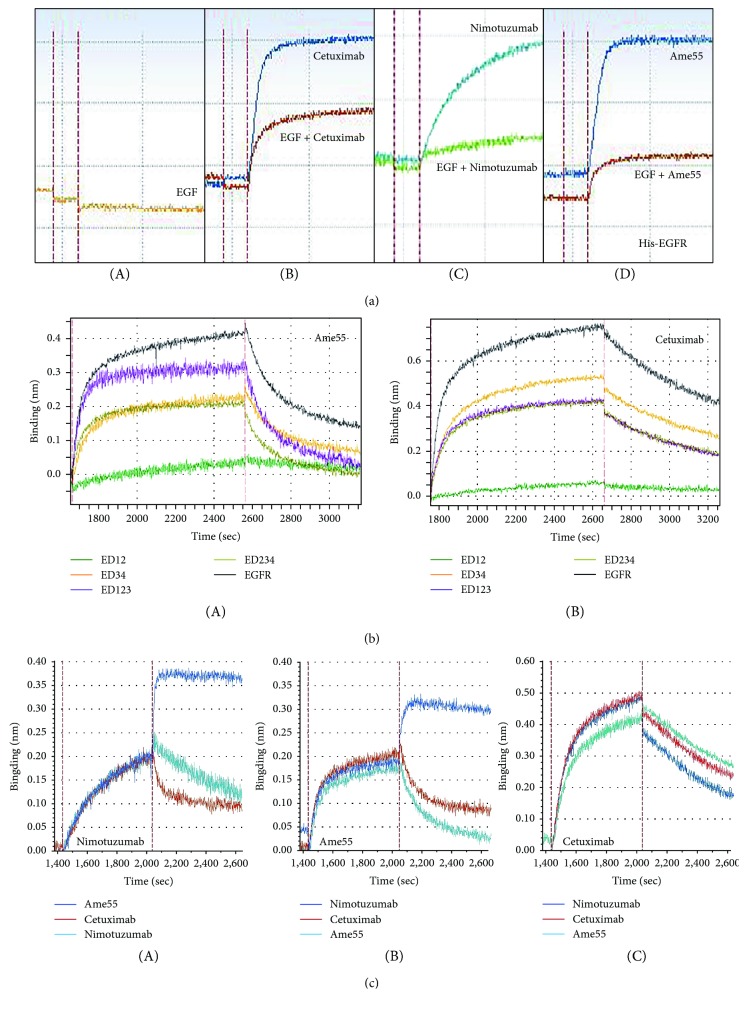
Ame55 shares different epitopes with cetuximab. (a) Ame55's binding with EGFR could be inhibited by EGF. SA sensor surfaces were coated with Bio-rhEGFR, 100 nM antibodies were added, and 1 *μ*M EGF was applied to inhibit the interaction. Cetuximab and hR3 were controls. (b) Interaction of Ame55 with different domains of EGFR. AHC sensor surfaces were coated with antibodies, and 200 nM recombinant proteins of EGFR truncated domains were added. (c) Competitive binding test for nimotuzumab, Ame55, and cetuximab. SA sensor surfaces were coated with first mAbs (nimotuzumab, Ame55, and cetuximab, separated in an A/B/C test), and then we loaded 200 nM his-EGFR to saturate binding; then, 500 nM of the three second antibodies were used to compete. The descent of the response means an overlapping between the epitopes of the two antibodies, whereas the elevation of the response means less overlap.

The EGFR domain III (D3) is the ligand binding site. Most antibodies inhibited EGFR via binding it, but their epitopes are different [17, 24, 25]. Here, we analyzed Ame55's binding domain with a ForteBio Octet QKe System (Pall ForteBio Corporation, Menlo, CA) as previously described [22]. The results indicated that Ame55 binding to EGFR could be inhibited by EGF in a similar manner to cetuximab and nimotuzumab (Figure 2(a)). Ame55 could bind with ED123, ED234, and ED34, but not with ED12, indicating that the main epitope was within domain III (Figure 2(b)). The competitive binding test indicated that (1) Ame55 could not inhibit the binding of nimotuzumab with EGFR, but could inhibit that of cetuximab; (2) nimotuzumab could not inhibit Ame55 binding to EGFR, but could inhibit that of cetuximab; and (3) cetuximab could inhibit both of them (Figure 2(c)). These results indicated that Ame55 has an epitope different from nimotuzumab (no overlapping), whereas cetuximab has an epitope overlapping in both Ame55 and nimotuzumab.

### 3.4. Ame55 Could Inhibit Tumor Growth Effectively in a Xenograft Model

**Figure 3:**
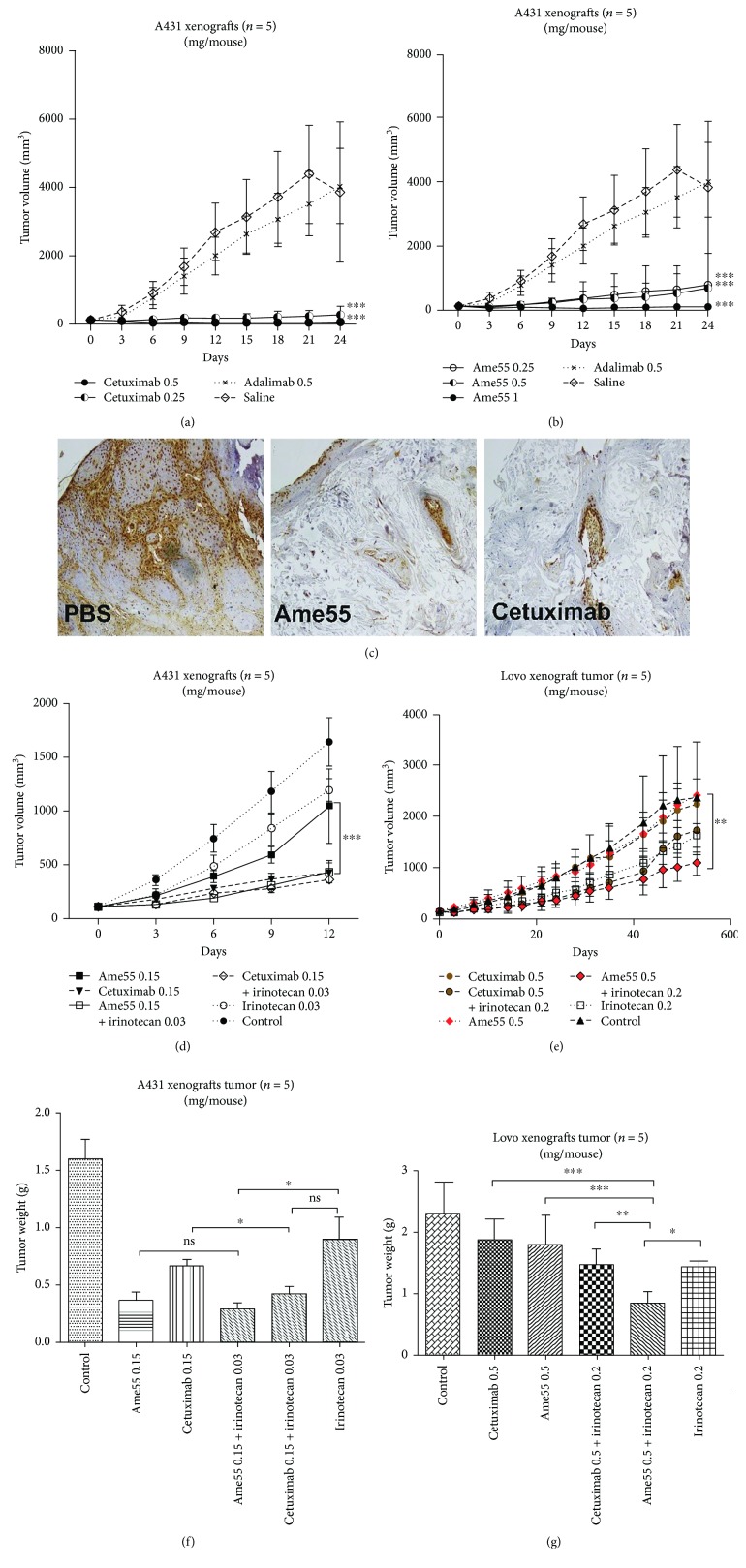
Antitumor effect of Ame55 alone or combined with irinotecan (CPT-11) in A431 and Lovo cell xenografts. Mice were injected and treated as indicated in Materials and Methods with (a) cetuximab (0.25, 0.5 nM); (b) Ame55 (0.25, 0.5, and 1 nM); (c) IHC analysis of PCNA expression for tumor cell proliferation inhibition in xenograft tumors (0.5 nM); (d-f) A431 cell xenograft mice (*n* = 5) were treated as described in Materials and Methods; (e-g) Lovo cell xenograft mice were treated as described in Materials and Methods.

Ame55's capability of inhibiting tumor growth was assessed in an A431 xenograft nude mouse model. After 24 days of twice-weekly antibody treatment, Ame55 (with doses of 0.25, 0.5, and 1 mg) significantly suppressed tumor growth (with inhibition rates of 82.04% for 0.25 mg, 85.38% for 0.5 mg, and 100.09% for 1 mg, respectively) compared to negative controls (Figure 3(a)). This inhibition ability was slightly lower than that of cetuximab (doses of 0.25 and 0.5 mg, with the corresponding inhibition rates of 95.93% and 101.48%, respectively) (Figure 3(b)). Tumor cell proliferation suppression was confirmed with PCNA immunohistochemical staining, showing that both Ame55 and cetuximab significantly decreased proliferating A431 tumor cell amount (Figure 3(c)).

### 3.5. A Significant Improvement of Antitumor Effects of Ame55 When Combined with Irinotecan

The antitumor effect of Ame55 when combined with irinotecan was tested in A431 and Lovo cell xenograft nude mouse models. In the A431 xenograft tumor model, the tumor inhibition potency of Ame55 plus irinotecan was significantly improved. In the Lovo cell xenograft tumor model, Ame55 combined with irinotecan was much more efficacious than irinotecan alone or irinotecan plus cetuximab. However, Ame55 and cetuximab had minimal efficacy when used alone, and cetuximab plus irinotecan had almost no improvement when compared with the irinotecan treatment alone in the two models (Figures 3(f) and 3(g)).

**Figure 4:**
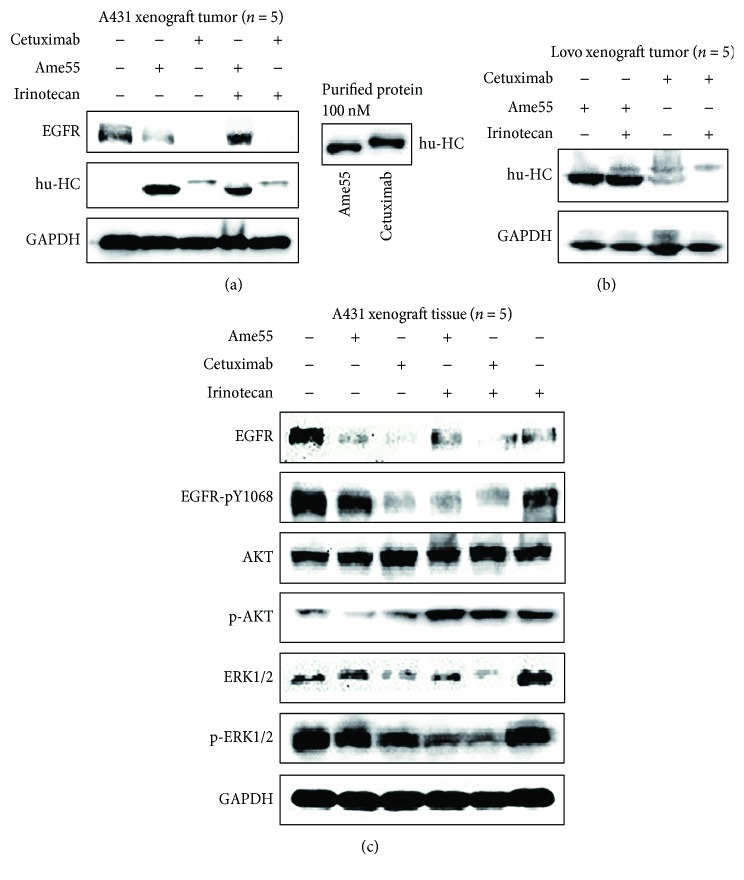
Ame55 accumulated in cells and tissues. (a) Antibody contents of Ame55 and C225 in A431 xenograft tumor tissues, with purified proteins as controls for the second antibody. (b) Antibody contents of Ame55 and C225 in Lovo xenograft tumor tissues. (c) Content of EGFR, pEGFR (Y1068), AKT, pAKT, ERK, and pERK by a single-dose antibody or combined treatment with irinotecan in A431 xenograft tumor tissues.

In addition, downstream EGFR signal pathways in tumor tissues were assessed. As shown in Figure 4(c), when Ame55 was combined with irinotecan, the changes in EGFR signal pathways were similar to those of cetuximab plus irinotecan. An accumulation of IgG1 at the tumor site was noted. An enhancement of p-AKT and a reduction of EGRFR-pY1068 and p-ERK1/2 were also noted, which indicated a stronger inhibition of the EGFR pathway when mAbs were combined with irinotecan. However, there were no significant changes for the EGFR-pY1068, p-AKT, p-ERK1/2, and Grb2, when either Ame55 or cetuximab was used alone (Figure 4(c)).

### 3.6. Ame55 Concentrates in EGFR Positive Tumor Tissues

We measured EGFR and antibody levels in the A431 cell xenograft nude mouse model when combining the antibody with irinotecan. Detection of antibody heavy chain (hu-HC) could reflect the IgG1 content in mouse tumors. In our experiments, Ame55 partially inhibited EGFR activity, and the concentration of Ame55 was much higher (5.4–6.4 folds) than that of cetuximab at the same dose level in high-EGFR-expressing A431 cells (Figure 4(a)). The EGFR level was decreased after the treatment of Ame55 alone and cetuximab alone, while it was not decreased in the Ame55 plus irinotecan group. Also, no significant change in EGFR level was observed in the cetuximab plus irinotecan group. In irinotecan-insensitive Lovo cells with medium EGFR expression, the concentration of Ame55 was also much higher (1.8–10.1 folds) than that of cetuximab at the same dose (Figure 4(b)). Interestingly, cetuximab concentration was decreased after the combination treatment with irinotecan, but Ame55 concentration was not affected by the combination.

### 3.7. No Toxicity of Ame55 in Cynomolgus Monkeys

We conducted the toxicity test of Ame55 in cynomolgus monkeys. No Ame55-related toxicity was noted when it was administrated to cynomolgus monkeys via intravenous infusion up to 300 mg/kg. All of the monkeys survived at the end of the study without any Ame55-related adverse findings in clinical observations, body weight, body temperature, hematology, coagulation, clinical chemistry, electrocardiogram, or gross necropsy. Therefore, the maximum tolerated dose (MTD) is equal to or greater than 300 mg/kg.

## 4. Discussion

In the current study, we generated a novel anti-EGFR antibody, Ame55, a fully human IgG1 mAb. Ame55 has a moderate avidity (1.7 nM) between that of cetuximab (0.46 nM) and h-R3 (7.6 nM) and showed much lower toxicity than these other antibodies in cynomolgus monkeys. The antitumor activity of Ame55 was good *in vivo*, and the tumor inhibition efficacy of Ame55 is better than that of cetuximab when combined with irinotecan in Lovo cell xenograft models.

As we expected, Ame55 showed low toxicity in cynomolgus monkeys on an acute toxicity study via a single intravenous infusion up to 300 mg/kg, which was 3 times the highest nominal dose of cetuximab [13]. There were no findings of biological significance. The low affinity and toxicity of Ame55 prompted us to improve the sequence and structure of Ame55, so that we could get a new molecular which has a higher affinity with EGFR to achieve better efficacy without increasing toxicity.

Beyond the lower affinity which is associated with low toxicity, Ame55 performed with antitumor efficacy similar to that of cetuximab *in vivo*. The tumor inhibition rate of Ame55 was 85.38% compared with cetuximab at 101.48% at a dose of 0.5 mg/mouse. Unexpectedly, there was a significant “triple jump” with tumor depression activity between *in vitro* and *in vivo* experiments. *In vitro* experiments showed lower potency of antitumor capacity, including cell proliferation inhibition (A431/DiFi cells), phase-G1 cell cycle blocking (DiFi cells), apoptosis stimulation (A431 cells), invasion (HaCaT cells), and migration (A431 cells) (see Supplemental Figures 3(a)–(c)), whereas the *in vivo* study showed comparable capacity of tumor depression with cetuximab in A431 and Lovo cells. To our surprise, Ame55 plus irinotecan performed more efficaciously than did cetuximab plus irinotecan or irinotecan alone in Lovo xenograft models that had lower EGFR expression levels and half-resistance to cetuximab. Ame55 also showed strong synergistic antitumor activity in combination with irinotecan in A431 xenograft mouse models.

In general, high efficacy accompanies high toxicity when targeting EGFR, which is widely expressed in epithelial tissues such as skin and gastrointestinal tissues. Therefore, the low toxicity of Ame55 was attributed to its relatively low affinity to EGFR, whereas the low effectiveness *in vitro* unexpectedly did not correspond to the *in vivo* antitumor efficiency. To explore the reason for this contradiction, we compared effects of Ame55 and cetuximab on the content and behavior of the anti-EGFR pathway. The results showed that Ame55 significantly accumulated in the tumor tissue following multiple administrations (about 5.4–6.4 folds compared to cetuximab in A431 xenograft tumors and 1.8–10 folds in Lovo xenograft tumors; see Figure 4(a)). This indicates that Ame55 concentrated and accumulated in tumors *in vivo*.

It was also a triple jump of Ame55 on pathway inhibition. Firstly, Ame55 has far less *in vitro* capacity than cetuximab in terms of tumor cell proliferation inhibition, apoptosis promotion, cell cycle arrest, migration inhibition, and anchoring growth inhibition (Supplemental Figure 3) and has lower suppression of EGFR and its downstream phosphorylation pathway. Secondly, in antibody-treated A431 cell xenograft tumor tissues, Ame55 has increased inhibition of the EGFR pathway, including total EGFR, pEGFR Y1068, and its downstream pAKT and pERK1/2, but the inhibition effect is still less than that of cetuximab. This suggests that the tumor tissue accumulation of Ame55 is helpful for EGFR pathway inhibition, but the role was still limited. Thirdly, after coadministration of irinotecan, the significant reduction of EGFR-pY1068 and pERK1/2 by Ame55 was accomplished just as well as by cetuximab. It is suggested that the inhibitory effect of Ame55 can be enhanced by irinotecan partially through the EGFR pathway inhibition mechanism.

It was reported that there were slight differences in epitopes between cetuximab and necitumumab. The latter has slightly less affinity to EGFR but superior tumor inhibition effect in NSCLC when compared to cetuximab. A computer modeling data demonstrated that the epitope recognized by nimotuzumab on the EGFR strongly overlapped with that of cetuximab [11]; our competition assay also showed the same phenomenon for cetuximab and nimotuzumab, and there was also an overlap between the epitopes of Ame55 and cetuximab. However, the data showed no overlapping between Ame55 and nimotuzumab (Figure 2(c)). These data indicate that Ame55 might have a different mechanism of action, and we should perform further investigation on it.

We believe that the accumulation of antibodies in the tumor site could bring a stronger antibody-dependent cell-mediated cytotoxicity (ADCC) effect. We especially showed in the irinotecan-Ame55 combinational treatment that this may make up for Ame55's low affinity and lack of pathway inhibition, thus killing tumor cells directly. ADCC is carried out through NK cells, macrophages, eosinophils, DC cells, etc. Although there are T/B cell defects in nude mice, they still contain NK, DC, and other immune cells and are still able to show a considerable role for the ADCC effect. Therefore, we believe that in the human body, where the immune system is intact, the accumulation of Ame55-induced ADCC effect would be more obvious than that in the ordinary nude mouse model.

Irinotecan, a broad-spectrum DNA topoisomerase I inhibitor, has been used as a first-line treatment in combination with 5-Fu/leucovorin for metastatic colorectal cancer [26]. ATP-binding cassette (ABC) transmembrane transporters such as P-glycoprotein (P-gp) and multidrug resistance-associated protein (MRP) contribute to the mechanisms of irinotecan resistance. Many tyrosine kinase inhibitors (TKI) such as EGFR TKIs have been reported as ABC inhibitors (chemosensitizers) [27]. Cetuximab has also been reported to increase the density of irinotecan and of its active metabolite SN-38 in colorectal carcinoma [28]. In our study, the efficacy of Ame55 combined with irinotecan in irinotecan-resistant Lovo cells was attributed to the accumulation of Ame55 and EGFR related irinotecan-resistant reversion. Nevertheless, it needs more investigations to answer the question on why irinotecan decreases concentration of cetuximab while it does not impact the concentration of Ame55.

In conclusion, we generated an EGFR-directed fully human antibody with low toxicity that can inhibit squamous cell carcinoma and colon cancer tumor growth in xenograft mice. The good therapeutic capacity of Ame55 combined with irinotecan was probably an integrated result of better penetration in tumor tissue, high accumulation in tumor tissue, resistance to degradation, enhancement of ADCC, and the unique EGFR-binding epitope. These characterisations of Ame55 make it have good potential for further clinical research and application.

## Figures and Tables

**Table 1:** Equilibrium dissociation constant (KD) values for affinity, avidity, or binding activity parameters of cetuximab and Ame55 binding to different EGFRs.

Antigen	His-rEGFR	Fc-rEGFR	A431 cells
Method	Biacore 3000		Cell ELISA
Cetuximab (IgG)	1.24 nM	BDL	0.46 nM
Cetuximab (Fab)	ND	1.39 nM	ND
Ame55 (IgG)	468 nM	0.23 nM	1.7 nM
Ame55 (Fab)	ND	931 nM	ND
Nimotuzumab (IgG)	ND	ND	7.6 nM

Notes: ND: not detected; BDL: below the detectable limit (0.1 nM).

## Data Availability

All data used for this project are publicly available and accessible online. We have annotated the entire data building process and empirical techniques presented in the paper.

## Supplementary Materials

Supplementary Materials Supplemental Figure 1: Biacore affinity analysis of Ame55 and cetuximab. (A) Ame55 binding to His-EGFR; (B) Ame55 binding to Fc-EGFR; (C) cetuximab binding to his-EGFR; (D) cetuximab binding to Fc-EGFR. Supplemental Figure 2: Ame55 was less efficacious at blocking cell proliferation and apoptosis. (A) Inhibition of A431 cells after treatment with Ame55 and cetuximab (0.12 to 15 *μ*g/mL) for 48 h (inhibition of DiFi cells treated the same as A431 cells). (B) Flow cytometry of A431 cells after PBS, cetuximab, or Ame55 coincubation for 2 h. (C) A431 cell apoptosis after treatment with antibodies for 48 h. (D) Molecular events in A431 and DiFi cells treated with indicated antibodies. Cell lysates (2 *μ*g total protein) were immunoblotted. Supplemental Figure 3: Ame55 inhibits migration but is less efficacious for blocking invasion compared to cetuximab. (A) Ame55 inhibited anchorage-independent HaCaT cell growth. Cells were exposed to 20, 100, and 400 nM Ame55, cetuximab, or adalimumab with EGF for 3 weeks. (A-2/3/4) Quantification of colonies from triplicate samples. Data are means ± SD, ^∗^ *p* < 0.05; ^∗∗∗^ *p* < 0.005 compared to EGF alone. (B) A scratch wound assay of A431 cells with 400 nM Ame55 and cetuximab for 48 h. (B-2) Quantification of relative length; representative experiments are shown in triplicate along with SD. (C-1) Ame55 and cetuximab inhibited cell migration in A431 cells. Magnification, 60x. (C-2) Quantification of cell numbers by relative area; representative experiments are shown in triplicate along with SD. ^∗∗∗^ *p* < 0.005 compared to EGF alone (D). Supplemental Figure 4: body weight of xenograft tumor mice on Figure 3. (A) Body weight of cetuximab-treated A431 cell xenograft mice described in Figure 3a. (B) Body weight of Ame55-treated A431 cell xenograft mice described in Figure 3b. (C) Body weight of antibodies combined treated A431 cell xenograft mice described in Figure 3d. (D) Body weight of antibodies combined treated Lovo cell xenograft mice described in Figure 3e. For (A-D), data are means ± SD. No statistical significant had been found.
