# Supplementary material for: A Novel Anti-EGFR mAb Ame55 with Lower Toxicity and Better Efficacy than Cetuximab When Combined with Irinotecan
Source: J Immunol Res. 2019 Jan 13;2019:3017360. doi: 10.1155/2019/3017360 (PMC6348820; doi:10.1155/2019/3017360)
Supplement: Supplementary Materials — Supplemental Figure 1: Biacore affinity analysis of Ame55 and cetuximab. (A) Ame55 binding to His-EGFR; (B) Ame55 binding to Fc-EGFR; (C) cetuximab binding to his-EGFR; (D) cetuximab binding to Fc-EGFR. Supplemental Figure 2: Ame55 was less efficacious at blocking cell proliferation and apoptosis. (A) Inhibition of A431 cells after treatment with Ame55 and cetuximab (0.12 to 15 μg/mL) for 48 h (inhibition of DiFi cells treated the same as A431 cells). (B) Flow cytometry of A431 cells after PBS, cetuximab, or Ame55 coincubation for 2 h. (C) A431 cell apoptosis after treatment with antibodies for 48 h. (D) Molecular events in A431 and DiFi cells treated with indicated antibodies. Cell lysates (2 μg total protein) were immunoblotted. Supplemental Figure 3: Ame55 inhibits migration but is less efficacious for blocking invasion compared to cetuximab. (A) Ame55 inhibited anchorage-independent HaCaT cell growth. Cells were exposed to 20, 100, and 400 nM Ame55, cetuximab, or adalimumab with EGF for 3 weeks. (A-2/3/4) Quantification of colonies from triplicate samples. Data are means ± SD, ∗ p < 0.05; ∗∗∗ p < 0.005 compared to EGF alone. (B) A scratch wound assay of A431 cells with 400 nM Ame55 and cetuximab for 48 h. (B-2) Quantification of relative length; representative experiments are shown in triplicate along with SD. (C-1) Ame55 and cetuximab inhibited cell migration in A431 cells. Magnification, 60x. (C-2) Quantification of cell numbers by relative area; representative experiments are shown in triplicate along with SD. ∗∗∗ p < 0.005 compared to EGF alone (D). Supplemental Figure 4: body weight of xenograft tumor mice on Figure 3. (A) Body weight of cetuximab-treated A431 cell xenograft mice described in Figure 3a. (B) Body weight of Ame55-treated A431 cell xenograft mice described in Figure 3b. (C) Body weight of antibodies combined treated A431 cell xenograft mice described in Figure 3d. (D) Body weight of antibodies combined treated Lovo cell xenograft mice describe [file 3017360.f1.pdf]

Supplemental data

1.1 Reagents, antibodies, and plasmids

Cetuximab was provided by Merck (Darmstadt, Germany), and adalimumab was purchased from Abbott (Chicago, IL). Antibodies against EGFR (pY1068\1173) /AKT /pAKT /ERK /pERK /Grb2 /GAPDH were purchased from Bioworld Technology (Nanjing, China), anti-EGFR antibody (SC-03) was from Santa Cruz (Paso Robles, CA), HRP-tag anti-M13KO7 antibody was from GE (Uppsala, Sweden). PCNA antibody for IHC was purchased from ZSGB-Bio (Beijing, China). Trans-5α, Trans1-Blue was from Transgene (Beijing, China). Fusion protein hFc-EGFR, His-EGFR and fully synthetic human scFv phage displayed libraries were constructed by our laboratory.

1.2 Screening of fully synthetic human scFv, IgG1 construction and expression

Phage displayed libraries were prepared according to recombinant phage selection module protocol Cat. #XY-040-00-05 (Pharmacia, Stockholm, Sweden). After 3 rounds of selection, single clones were screened by ELISA with BSA as a negative control. VH and VL genes of immunopositive scFvs were cloned into expression vector pAbG1 using restriction enzyme sites for heavy chain: *AflII* and *NheI*, and for light chain: *BsrGI* and *HindIII*. Heavy chain and light chain expression vectors were used to co-transfect FreeStyleTM 293-F cells (Invitrogen, Carlsbad, CA) for instantaneous expressions. Supernatants containing IgG1 were collected and purified with a ProteinA 1 mL column (GE Healthcare, Uppsala, Sweden). Then, 10% SDS-PAGE reducing gel was used confirm antibody purity.

1.3 Cell ELISA

A431 cells were seeded in 96 well-plates, from 0.5×10^4/well to 4×10^4/well, and were cultivated overnight at 37°C, 5% CO_2. Antibodies were diluted to 10 μg/mL with PBS containing 2% FBS and serially two-fold diluted for 8 gradients. After 1 h on ice, plates were washed with PBST. HRP-conjugated goat anti-human IgG (Sigma-Aldrich, A0170) was added and incubated for 30 min on ice. After washing, o-phenylenediamine dihydro-chloride (OPD) substrate was added, and optical density (OD) was read at 492 nm with 630 nm as a reference using a microplate reader (Thermo Multiskan MK3, Piedmont, SC).

1.4 Binding specificity analysis by ELISA

For the binding specificity assay, 200 ng His-EGFR, VEGF, IL-6, BSA, CD4, P-selectin, Aβ-T (all proteins were expressed by our laboratory) and PBS were coated in 96-well plates (Costar 9018, Corning, NY) in PBS buffer and incubated 4°C overnight, with 5% fat-free powdered milk in PBS with 0.1% Tween20 were used for blocking for 30min at 37°C, Cetuximab and Ame55 were used as primary antibodies and incubated for 1 h at 37°C, after 3 washings, HRP-goat anti-human IgG (ZSGB-BIO, Beijing, China) were used as a second antibody and incubated for 30 min at 37°C. Plates were developed with OPD and OD was read at 492/630 nm using a 470 microplate reader (Thermo Multiskan MK3, Piedmont, SC).

1.5 Immunofluorescence assay

For EGFR-antibody binding assay, 10000 of exponential phased growth CHO, Lovo and A431 cells were seeded on coverslips in 24-well tissue culture plates. After overnight culture, cells were washed with PBS containing 4% paraformaldehyde (Beijing Chemical Works, Beijing, China) and 0.1% triton X-100 (Sigma, St. Louis, MO), and washed with PBS at room temperature. Cells were then blocked with 5% goat serum in PBS for 30 min, washed three times with PBS, and incubated with 50 μg/mL Ame55 or cetuximab, for 2 h at room temperature. PBS was a negative control. Cells were then washed three times with PBS and incubated with FITC-labeled goat anti-mouse IgG (ZSGB-BIO, Beijing, China) diluted at 1:200 in PBS for 30 min. Cells were rinsed in PBS three times and visualized under a microscope with 100× zoom in (Nikon Xi-80, Tokyo, Japan).

1.6 CCK8 proliferation assay

A CCK-8 Kit (Dojindo Laboratories, Rockville, MD) was used to measure cell growth of A431 and DiFi cells. Cells (10,000 cells/per well) were seeded in 96-well plates. Antibodies were added and incubated at 37°C in 5% CO_2 for 72 h. CCK-8 solution (12 μL) was added to 100 μL culture media, and OD was read at 450/630 nm. Three independent experiments were performed.

1.7 FACS analysis of cell cycle and apoptosis

After 24 h treatment with 30 μg/mL Ame55 or cetuximab, A431 cells were harvested, washed with PBS, and fixed in 70% ethanol overnight. Cells were washed with PBS twice, and stained with propidium iodide buffer (50 μg/mL PI Basle, Switzerland) with 20 μg/mL RNase) for 25 min at 37°C in the dark. Cell cycle phase distribution was assessed with flow cytometry (Cytomics FC 500, Beckman Coulter). Cells in each phase were analyzed by using

Modifit LT.

An Alexa Fluor® 488 annexin V/Dead Cell Apoptosis Kit (Invitrogen,V13241, Paisley, UK) was used to quantify apoptosis. Serum-starved cells were treated with 30 μg/mL Ame55 or cetuximab for 24 h, harvested and resuspended with annexin binding buffer, then 5μL Component A and 1 μL 100 μg/mL PI was added to 100 μL cell sample. Samples were incubated in the dark for 15 min at room temperature, and 400 μL annexin binding buffer was added. Cells were analyzed with FACS cytometry (Cytomics FC 500, Beckman Coulter) and Modifit LT.

1.8 Anchorage-independent transformation assay

The effect of antibodies on EGF-induced cell transformation was investigated in HaCaT cells. Cells (10,000 cells/well in 6-well plates) were exposed simultaneously to different concentrations (20, 100, 400 nM) of Ame55 or cetuximab with EGF (10 ng/mL) in 0.35% agar 1640 containing 10% FBS over 0.6% agar 1640 containing 10% FBS for 3 weeks. Three independent experiments were performed, and cell colonies were counted in 5 random fields for each culture dish, scored and statistically assessed.

1.9 *In vitro* wound healing assay

Approximately 1.2 × 10^6 cells were seeded on Falcon 6-well tissue culture plates. After 12 h, confluent cells were incubated in serum-free medium for 24 h (~80% plate coverage). Scratch wounds were created by scraping the confluent monolayers with a sterile 200 μL pipette tip to create an ~1.0-mm gap. Four parallel scrapes per well at the same position were made and cells dislodged by scraping were removed by washing cultures with serum-free medium. Cells were treated with cetuximab or Ame55 at 400 nM. Migration of the cells into the scratch was observed at 0, 12, and 48 h after scraping (200× magnification).

1.10 Transwell migration assay

Cells (1 × 10^5) were suspended in 200 μL 1640 medium without serum and placed in (8 μm-pore size; Millipore, Merck, Darmstadt, Germany) of a companion plate (12-well plate, Corning, Corning, NY) with a prewarmed culture medium containing 10% FBS in the well. Then, 400 nM cetuximab or Ame55 antibodies were added the insert chamber and mixed. PBS was a control. Cells were incubated for 18 h at 37°C in 5% CO_2. The top of the filter was gently wiped with a cottonswab to remove non-migrated cells. Cell migration was measured by staining cells with 1% crystal violet and photographing cells under a light microscope (400× magnification).

1.11 Immunohistochemical Analysis

To assess angiogenesis and cell proliferation in tumors, formalin-fixed paraffin-embedded

tumor tissues were immunostained with a monoclonal mouse anti-human PCNA and S9001 IHC staining kit (ZSGB-BIO, Beijing, China). After deparaffinization and rehydration, the tissue sections were incubated with 3% hydrogen peroxide in methanol to quench endogenous peroxidase. Sections were blocked for 30 min with goat serum at recommended concentration and incubated overnight with primary antibody at 4°C. Sections were then washed with PBS and incubated with a biotinylated secondary antibody for 30 min, and finally incubated with HRP-conjugated streptavidin for 30 min. Products were visualized using a diaminobenzidine ZLI-9017 staining kit (ZSGB-BIO, Beijing, China) and counterstained in hematoxylin.

Supplemental Figure Legends

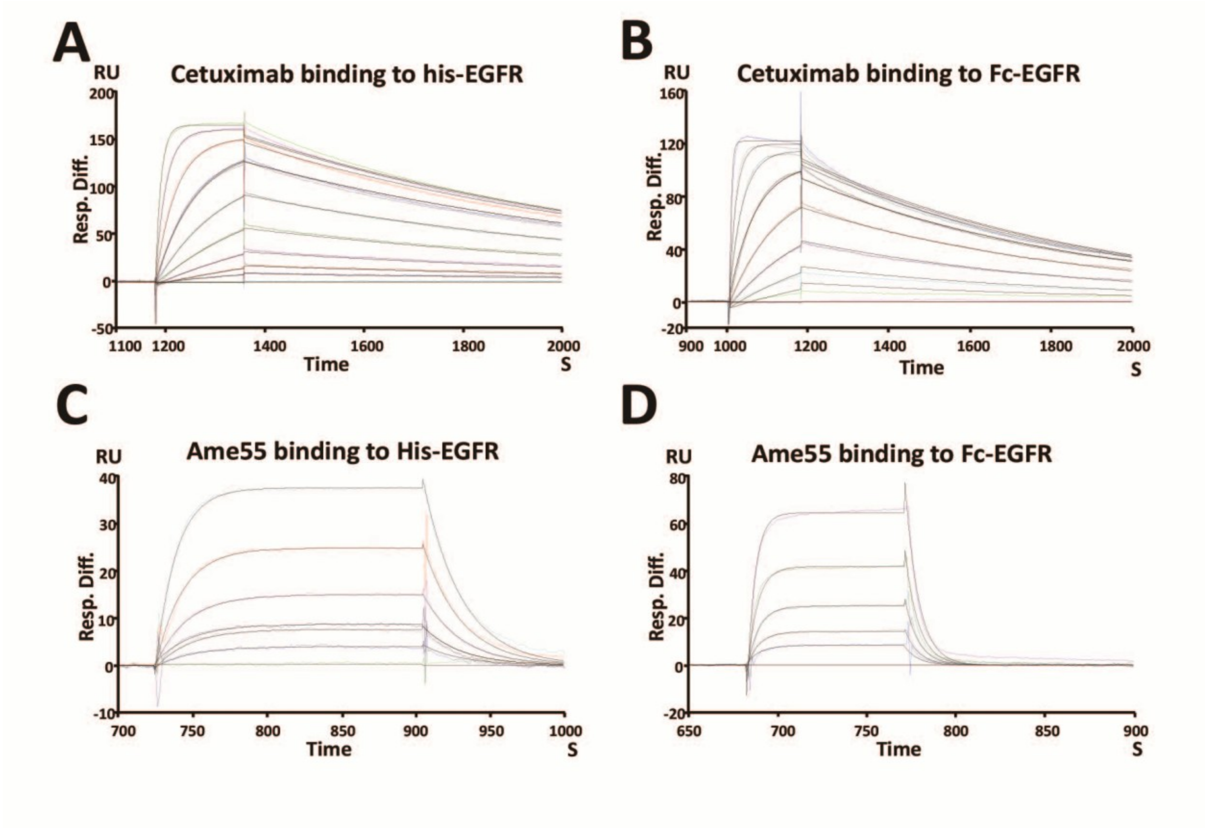

Supplemental Figure1: Biacore affinity analysis of Ame55 and cetuximab. A. Ame55 binding to His-EGFR; B. Ame55 binding to Fc-EGFR C. Cetuximab binding to his-EGFR; D. Cetuximab binding to Fc-EGFR.

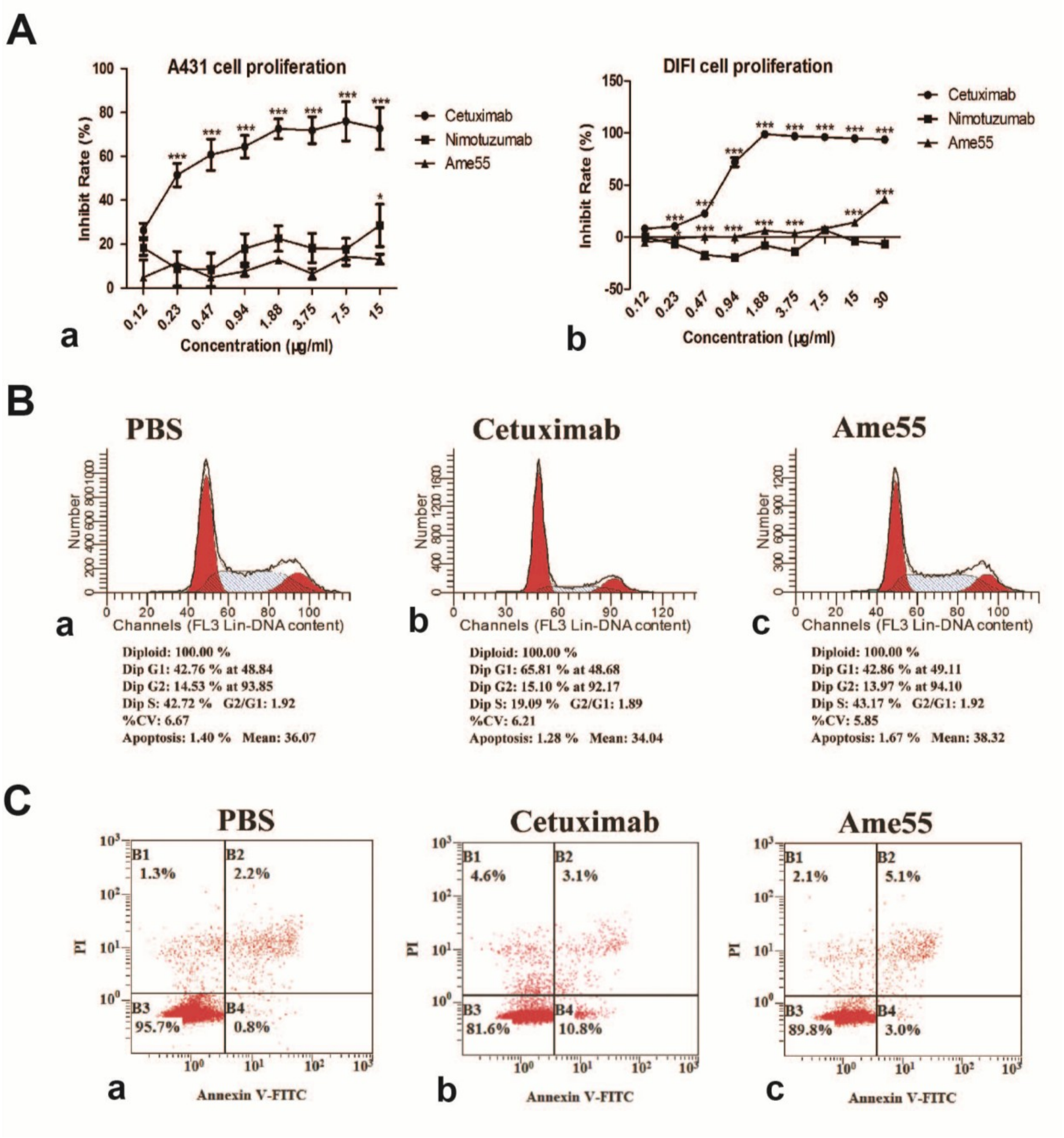

Supplemental Figure2. Ame55 was less efficacious at blocking cell proliferation and apoptosis. (A) Inhibition of A431 cells after treatment with Ame55 and cetuximab (0.12 to 15 μg/mL) for 48 h. (inhibition of DiFi cells treated the same as A431cells). (B) Flow cytometry of A431 cells after PBS, cetuximab or Ame55 co-incubation for 2 h. (C) A431 cell apoptosis after treatment with antibodies for 48 h. D Molecular events in A431 and DiFi cells treated with indicated antibodies. Cell lysates (2 μg total protein) were immunoblotted.

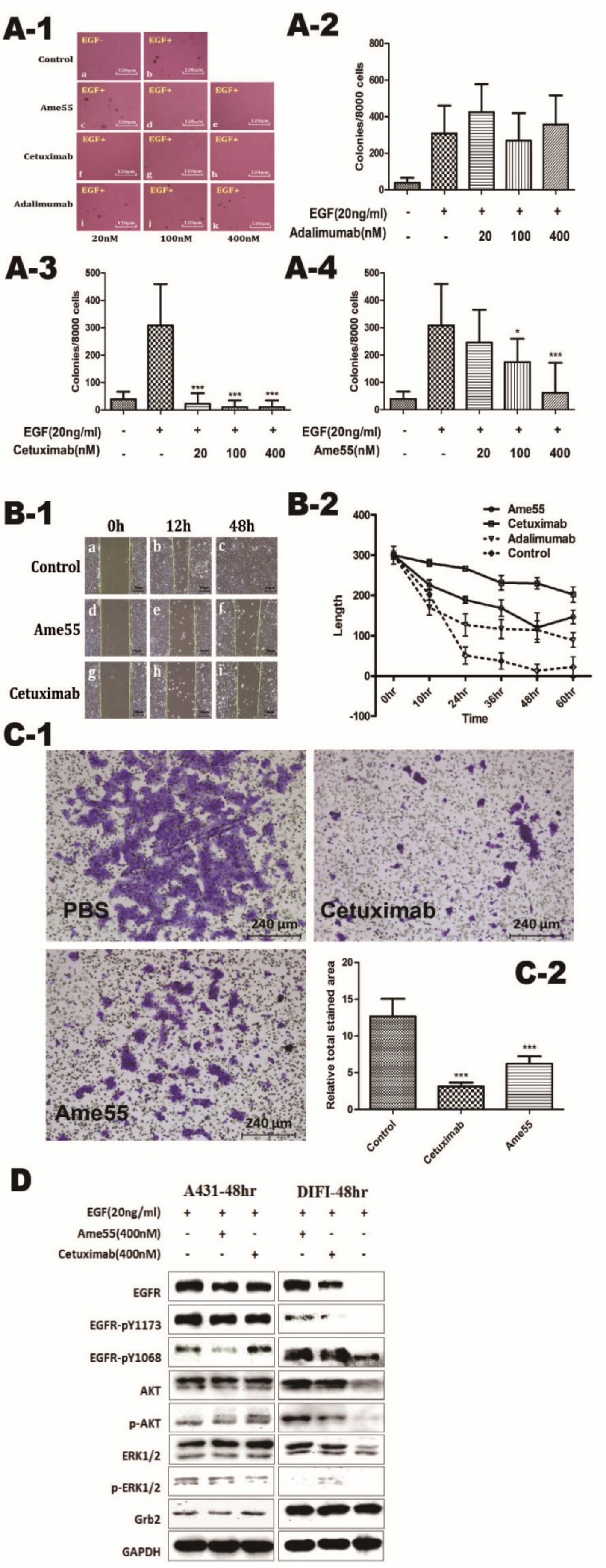
A-1
Control
Ame55
Cetuximab
Adalimumab
20nM
100nM
400nM
A-2
Colonies/8000 cells
EGF(20ng/ml)
Adalimumab(nM)
A-3
Cetuximab(nM)
A-4
Ame55(nM)
B-1
0h
12h
48h
B-2
Ame55
Cetuximab
Adalimumab
Control
Length
Time
C-1
PBS
Cetuximab
Ame55
240 μm
C-2
Relative total stained area
D
A431-48hr
DIFI-48hr
EGF(20ng/ml)
Ame55(400nM)
Cetuximab(400nM)
EGFR
EGFR-pY1173
EGFR-pY1068
AKT
p-AKT
ERK1/2
p-ERK1/2
Grb2
GAPDH

Supplemental Figure3. Ame55 inhibits migration but is less efficacious for blocking invasion compared to cetuximab. (A) Ame55 inhibited anchorage-independent HaCaT cell growth. Cells were exposed to 20, 100, 400 nM Ame55, cetuximab or adalimumab with EGF for 3 weeks. (A-2/3/4) Quantification of colonies from triplicate samples. Data are means ± S.D., * = $p < 0.05$; *** = $p < 0.005$ compared to EGF alone. (B) A scratch wound assay of A431 cells with 400 nM Ame55 and Cetuximab for 48 h. (B-2) Quantification of relative length, representative experiments are shown in triplicate along with SD. (C-1) Ame55 and cetuximab inhibited cell migration in A431 cells. Magnification, 60x. (C-2) Quantification of cell numbers by relative area, representative experiments are shown in triplicate along with SD. *** = $p < 0.005$ compared to EGF alone. (D)

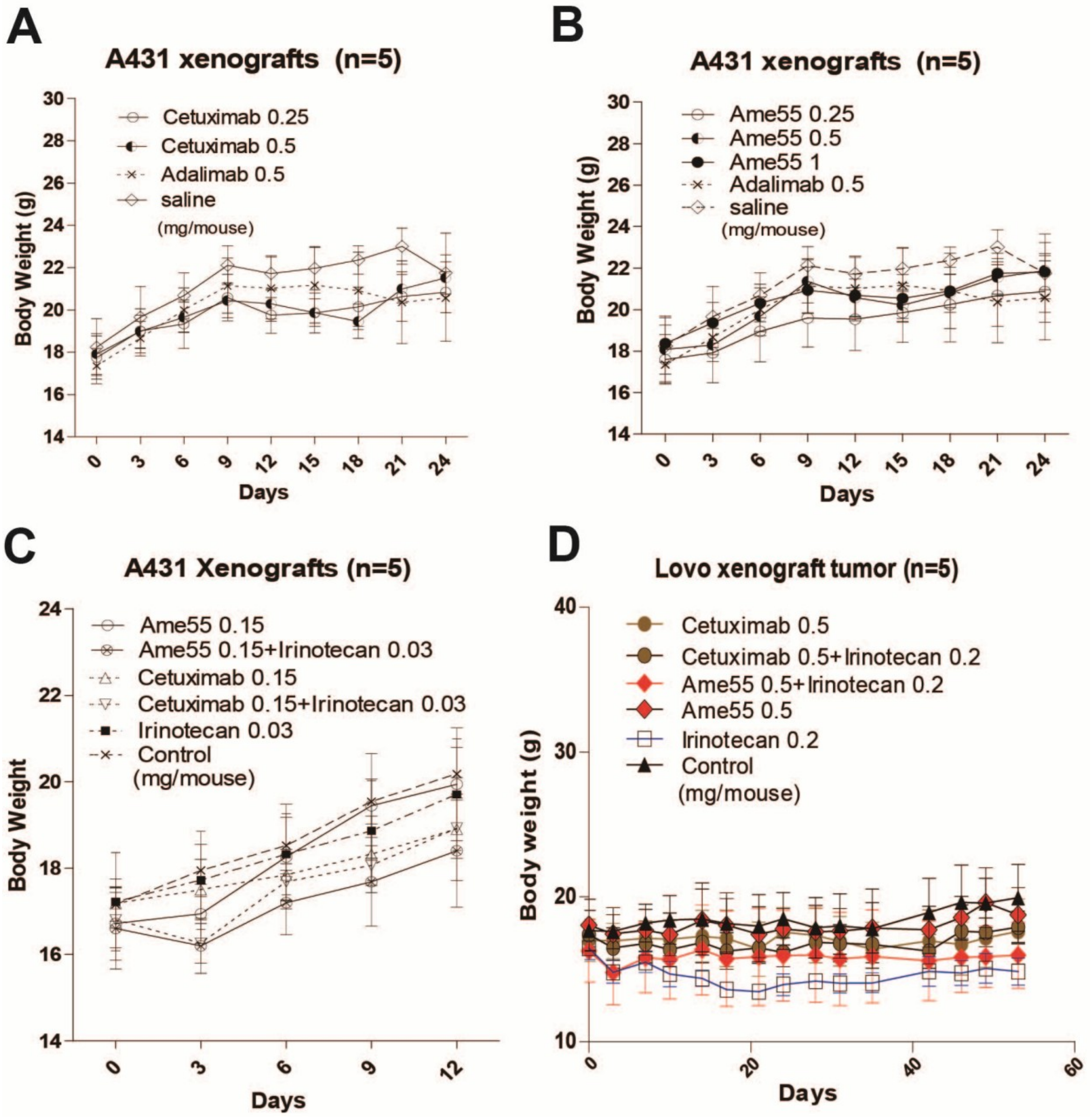

Supplemental Figure4. Body weight of xenograft tumor mic on Figure 5. (A)Body wight of Cetuximab treated A431 cell xenogfraft mice described in Figure5A. (B) Body wight of Ame55 treated A431 cell xenogfraft mice described in Figure5B. (C) Body wight of antibodies combined treated A431 cell xenogfraft mice described in Figure5D. (D) Body wight of antibodies combined treated Lovo cell xenogfraft mice described in Figure5F. For (A-D), Data are means ± S.D., No statistical significant had been found.

Supplemental Table.

Supplemental Table 1. Biacore affinity analysis of Ame55 and cetuximab

	**antigen**	**ka (1/Ms)**	**kd (1/s)**	**Rmax (RU)**	**KA (1/M)**	**KD (M)**	**Chi2**
**Ame55(Fab')**	hFc-EGFR[I]	4.19E+04	0.039	87	1.07E+06	**9.31E-07**	0.436
**Cetuximab(Fab')**	mFc-EGFR[I]	1.61E+06	2.24E-03	38.2	7.2E+08	**1.39E-09**	0.275
**Ame55(IgG)** [I]	his-EGFR	2.83E+05	0.133	194	2.14E+06	**4.68E-07**	1.59
**Cetuximab(IgG)** [I]	his-EGFR	9.01E+05	1.12E+03	157	8.03E+08	**1.24E-09**	2.16
**Ame55(IgG)**	Fc-EGFR[I]	4.21E+05	9.79.7E-05	691	4.33E+09	**2.31E-10**	64.8
**Cetuximab(IgG)**	Fc-EGFR[I]	1.1E+06	2.1E-07	1.24E+03	5.25E+12	**1.9E-13**[*]	150

[I]: immobile phase; [*]: The value is lower than detectable interval(<0.1 nM).
